# (*E*)-*N*′-(3-Eth­oxy-4-hy­droxy­benzyl­idene)-4-meth­oxy­benzohydrazide

**DOI:** 10.1107/S1600536812007374

**Published:** 2012-02-24

**Authors:** Hoong-Kun Fun, Premrudee Promdet, Jirapa Horkaew, Chatchanok Karalai, Suchada Chantrapromma

**Affiliations:** a X-ray Crystallography Unit, School of Physics, Universiti Sains Malaysia, 11800 USM, Penang, Malaysia; b Crystal Materials Research Unit, Department of Chemistry, Faculty of Science, Prince of Songkla University, Hat-Yai, Songkhla 90112, Thailand

## Abstract

In the mol­ecule of the title benzohydrazide derivative, C_17_H_18_N_2_O_4_, the dihedral angle between the benzene rings is 6.86 (11)°. The meth­oxy group of the 4-meth­oxy­phenyl fragment deviates slightly [C_methyl_—O—C—C = 10.0 (4)°] with respect to the benzene ring, whereas the eth­oxy group of the 3-eth­oxy-4-hy­droxy­phenyl fragment is is almost coplanar [C—O—C—C_methyl_ = 178.5 (2)°]. In the crystal, mol­ecules are linked by N—H⋯O, O—H⋯O and C—H⋯O hydrogen bonds into a two-dimensional network parallel to the *ab* plane. C—H⋯π inter­actions and C⋯O [2.980 (3) Å] short contacts are also observed.

## Related literature

For bond-length data, see: Allen *et al.* (1987 ▶). For hydrogen-bond motifs, see: Bernstein *et al.* (1995 ▶). For related structures, see: Fun *et al.* (2011 ▶); Horkaew *et al.* (2011 ▶); Promdet *et al.* (2011 ▶). For background and applications to benzohydrazide derivatives, see: Bedia *et al.* (2006 ▶); Loncle *et al.* (2004 ▶); Raj *et al.* (2007 ▶).
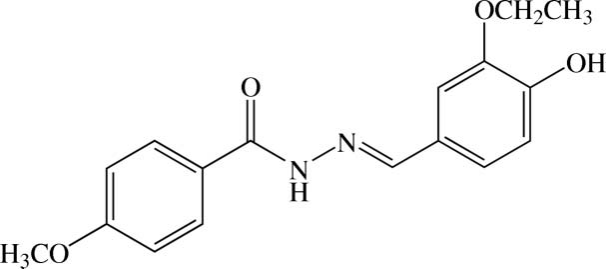


## Experimental

### 

#### Crystal data

C_17_H_18_N_2_O_4_
*M* *_r_* = 314.33
Orthorhombic, 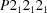

*a* = 5.0607 (9) Å
*b* = 11.086 (2) Å
*c* = 27.629 (5) Å
*V* = 1550.1 (5) Å^3^
*Z* = 4
Mo *K*α radiation
μ = 0.10 mm^−1^
*T* = 297 K
0.56 × 0.10 × 0.07 mm

#### Data collection

Bruker APEX DUO CCD area-detector diffractometer
Absorption correction: multi-scan (*SADABS*; Bruker, 2009 ▶) *T*_min_ = 0.948, *T*_max_ = 0.993
10252 measured reflections
2637 independent reflections
1921 reflections with *I* > 2σ(*I*)
*R*_int_ = 0.100

#### Refinement

*R*[*F* ^2^ > 2σ(*F* ^2^)] = 0.051
*wR*(*F* ^2^) = 0.154
*S* = 1.04
2637 reflections
210 parameters
H-atom parameters constrained
Δρ_max_ = 0.36 e Å^−3^
Δρ_min_ = −0.35 e Å^−3^

### 

Data collection: *APEX2* (Bruker, 2009 ▶); cell refinement: *SAINT* (Bruker, 2009 ▶); data reduction: *SAINT*; program(s) used to solve structure: *SHELXTL* (Sheldrick, 2008 ▶); program(s) used to refine structure: *SHELXTL*; molecular graphics: *SHELXTL*; software used to prepare material for publication: *SHELXTL* and *PLATON* (Spek, 2009 ▶).

## Supplementary Material

Crystal structure: contains datablock(s) global, I. DOI: 10.1107/S1600536812007374/rz2713sup1.cif

Structure factors: contains datablock(s) I. DOI: 10.1107/S1600536812007374/rz2713Isup2.hkl

Supplementary material file. DOI: 10.1107/S1600536812007374/rz2713Isup3.cml

Additional supplementary materials:  crystallographic information; 3D view; checkCIF report

## Figures and Tables

**Table 1:** Hydrogen-bond geometry (Å, °) *Cg*1 is the centroid of the C9–C14 ring.

*D*—H⋯*A*	*D*—H	H⋯*A*	*D*⋯*A*	*D*—H⋯*A*
O4—H1*O*4⋯O3	0.82	2.41	2.683 (2)	100
O4—H1*O*4⋯O1^i^	0.82	2.21	2.981 (2)	156
N1—H1*N*1⋯O1^ii^	0.90	2.10	2.994 (3)	172
C10—H10*A*⋯O4^iii^	0.93	2.55	3.462 (3)	168
C16—H16*B*⋯*Cg*1^i^	0.97	2.68	3.499 (2)	142

Symmetry codes: (i) 
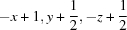
; (ii) 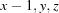; (iii) 
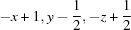
.

**Fig. 1.**
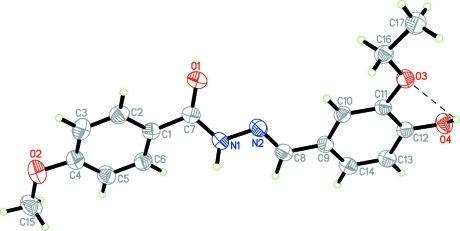
The molecular structure of the title compound, showing 45% probability displacement ellipsoids. Hydrogen bond are drawn as dashed line.

**Fig. 2.**
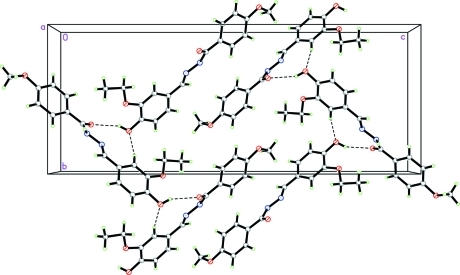
The crystal packing of the title compound viewed along the a axis. Hydrogen bonds are drawn as dashed lines.

## supplementary crystallographic information

### Comment

During the course of our on-going research on benzohydrazide derivatives, which have been reported to possess various biological properties such as antibacterial and antifungal (Loncle *et al.*, 2004), antitubercular (Bedia *et al.*, 2006) and antiproliferative (Raj *et al.*, 2007) activities, the synthesis and crystal structures of benzohydrazide derivatives have been reported (Fun *et al.*, 2011; Horkaew *et al.*, 2011; Promdet *et al.*, 2011). The title compound was synthesized and tested for its antioxidant and antibacterial activities and found to be inactive.

The molecule of the title benzohydrazide derivative (Fig. 1) exists in a *trans*-configuration with respect to the C8═N2 bond [1.286 (4) Å], as indicated by the torsion angle N1–N2–C8–C9 = -178.2 (2)°. The molecule is slightly twisted with the dihedral angle between the two benzene rings of 6.86 (11)°. The middle bridge fragment (O1/C7/N1/N2/C8) is planar with the torsion angle N2–N1–C7–O1 = 0.8 (4)° and the r.m.s. of 0.0360 (2) Å for the five non-H atoms. The mean plane through this bridge makes dihedral angles of 28.94 (15) and 26.51 (15)° with the C1–C6 and C9–C14 phenyl rings, respectively. The methoxy group of 4-methoxyphenyl is slightly twisted with respect to its bound benzene ring [torsion angle C15–O2–C4–C5 = 10.0 (4)°], whereas the ethoxy group of 3-ethoxy-4-hydroxyphenyl is co-planar with the torsion angle C11–O3–C16–C17 = 178.5 (2)°.

An intramolecular O4—H1O4···O3 hydrogen bond generates a S(5) ring motif (Bernstein *et al.*, 1995). Bond distances are of normal values (Allen *et al.*, 1987) and are comparable with those found in related structures (Fun *et al.*, 2011; Horkaew *et al.*, 2011; Promdet *et al.*, 2011).

In the crystal packing (Fig. 2), the molecules are linked by N—H···O and O—H···O hydrogen bonds and weak C—H···O interactions (Table 1) into a two-dimensional network paralell to the *ab* plane. The crystal is further stabilized by C—H···π weak interaction (Table 1). A C8···O4^iv^ [2.980 (3) Å: (iv) = -x, 1/2+y, 1/2+z] short contact was observed.

### Experimental

The title compound was prepared by dissolving 4-methoxybenzohydrazide (2 mmol, 0.30 g) in ethanol (10 ml). A solution of 3-ethoxy-4-hydroxybenzaldehyde (2 mmol, 0.30 g) in ethanol (10 ml) was then added slowly to the reaction. The mixture was refluxed for about 5 h and a white solid appeared. The mixture was then cooled to room temperature and filtered. Colourless needle-shaped single crystals of the title compound suitable for X-ray structure determination were recrystallized from methanol by slow evaporation of the solvent at room temperature after several days. M. p. 486-488 K.

### Refinement

All H atoms were positioned geometrically and allowed to ride on their parent atoms, with d(N-H) = 0.90 Å, d(O-H) = 0.82 Å, d(C-H) = 0.93 Å for aromatic and CH, 0.97 for CH_2_ and 0.96 Å for CH_3_ atoms. The *U*_iso_ values were constrained to be 1.5*U*_eq_(C, N, O) for methyl, hydroxy and amine H atoms and 1.2*U*_eq_(C) for the remaining H atoms. A rotating group model was used for the methyl groups. 1879 Friedel pairs were merged. Three outliers (2 1 1, 1 4 0, 3 2 12) were omitted for the final refinement.

### Crystal data

**Table:**

C_17_H_18_N_2_O_4_	*D*_x_ = 1.347 Mg m^−^^3^
*M**_r_* = 314.33	Melting point = 486–488 K
Orthorhombic, *P*2_1_2_1_2_1_	Mo *K*α radiation, λ = 0.71073 Å
Hall symbol: P 2ac 2ab	Cell parameters from 2637 reflections
*a* = 5.0607 (9) Å	θ = 1.5–30.1°
*b* = 11.086 (2) Å	µ = 0.10 mm^−^^1^
*c* = 27.629 (5) Å	*T* = 297 K
*V* = 1550.1 (5) Å^3^	Needle, colorless
*Z* = 4	0.56 × 0.10 × 0.07 mm
*F*(000) = 664	

### Data collection

**Table:**

Bruker APEX DUO CCD area-detector diffractometer	2637 independent reflections
Radiation source: sealed tube	1921 reflections with *I* > 2σ(*I*)
Graphite monochromator	*R*_int_ = 0.100
φ and ω scans	θ_max_ = 30.1°, θ_min_ = 1.5°
Absorption correction: multi-scan (*SADABS*; Bruker, 2009)	*h* = −7→7
*T*_min_ = 0.948, *T*_max_ = 0.993	*k* = −15→15
10252 measured reflections	*l* = −38→29

### Refinement

**Table:**

Refinement on *F*^2^	Primary atom site location: structure-invariant direct methods
Least-squares matrix: full	Secondary atom site location: difference Fourier map
*R*[*F*^2^ > 2σ(*F*^2^)] = 0.051	Hydrogen site location: inferred from neighbouring sites
*wR*(*F*^2^) = 0.154	H-atom parameters constrained
*S* = 1.04	*w* = 1/[σ^2^(*F*_o_^2^) + (0.0865*P*)^2^ + 0.0494*P*] where *P* = (*F*_o_^2^ + 2*F*_c_^2^)/3
2637 reflections	(Δ/σ)_max_ = 0.001
210 parameters	Δρ_max_ = 0.36 e Å^−^^3^
0 restraints	Δρ_min_ = −0.35 e Å^−^^3^

### Special details

**Table:**

Geometry. All esds (except the esd in the dihedral angle between two l.s. planes) are estimated using the full covariance matrix. The cell esds are taken into account individually in the estimation of esds in distances, angles and torsion angles; correlations between esds in cell parameters are only used when they are defined by crystal symmetry. An approximate (isotropic) treatment of cell esds is used for estimating esds involving l.s. planes.
Refinement. Refinement of F^2^ against ALL reflections. The weighted R-factor wR and goodness of fit S are based on F^2^, conventional R-factors R are based on F, with F set to zero for negative F^2^. The threshold expression of F^2^ > 2sigma(F^2^) is used only for calculating R-factors(gt) etc. and is not relevant to the choice of reflections for refinement. R-factors based on F^2^ are statistically about twice as large as those based on F, and R- factors based on ALL data will be even larger.

### Fractional atomic coordinates and isotropic or equivalent isotropic displacement parameters (Å^2^)

**Table:**

	*x*	*y*	*z*	*U*_iso_*/*U*_eq_	
O1	0.7066 (4)	0.1645 (2)	0.40465 (7)	0.0575 (5)	
O2	0.2255 (5)	−0.16566 (19)	0.57022 (7)	0.0623 (6)	
O3	0.5434 (4)	0.52128 (16)	0.19944 (6)	0.0450 (4)	
O4	0.2111 (4)	0.70975 (15)	0.20065 (7)	0.0499 (5)	
H1O4	0.2354	0.6755	0.1747	0.075*	
N1	0.2812 (5)	0.2287 (2)	0.40261 (8)	0.0445 (5)	
H1N1	0.1137	0.2022	0.4041	0.067*	
N2	0.3378 (5)	0.30448 (19)	0.36411 (8)	0.0465 (5)	
C1	0.4040 (5)	0.0790 (2)	0.46083 (9)	0.0390 (5)	
C2	0.5413 (6)	−0.0295 (3)	0.46673 (9)	0.0469 (6)	
H2A	0.6794	−0.0482	0.4458	0.056*	
C3	0.4754 (7)	−0.1084 (2)	0.50282 (9)	0.0518 (7)	
H3A	0.5660	−0.1810	0.5057	0.062*	
C4	0.2741 (6)	−0.0809 (2)	0.53512 (9)	0.0460 (6)	
C5	0.1377 (7)	0.0268 (3)	0.53087 (10)	0.0516 (7)	
H5A	0.0048	0.0464	0.5527	0.062*	
C6	0.2019 (6)	0.1050 (2)	0.49356 (10)	0.0471 (6)	
H6A	0.1079	0.1766	0.4902	0.057*	
C7	0.4803 (5)	0.1604 (2)	0.42070 (9)	0.0415 (5)	
C8	0.1489 (6)	0.3765 (2)	0.35246 (9)	0.0439 (6)	
H8A	−0.0064	0.3750	0.3705	0.053*	
C9	0.1701 (5)	0.4600 (2)	0.31206 (9)	0.0394 (5)	
C10	0.3601 (5)	0.4457 (2)	0.27571 (9)	0.0387 (5)	
H10A	0.4775	0.3812	0.2770	0.046*	
C11	0.3739 (5)	0.5275 (2)	0.23787 (8)	0.0363 (5)	
C12	0.2013 (5)	0.6265 (2)	0.23665 (9)	0.0374 (5)	
C13	0.0117 (6)	0.6400 (2)	0.27242 (9)	0.0438 (6)	
H13A	−0.1046	0.7049	0.2714	0.053*	
C14	−0.0044 (6)	0.5566 (2)	0.30982 (9)	0.0455 (6)	
H14A	−0.1331	0.5656	0.3336	0.055*	
C15	−0.0056 (8)	−0.1503 (4)	0.59944 (11)	0.0733 (10)	
H15A	−0.0236	−0.2179	0.6209	0.110*	
H15B	−0.1585	−0.1453	0.5790	0.110*	
H15C	0.0102	−0.0776	0.6180	0.110*	
C16	0.7123 (5)	0.4166 (2)	0.19693 (9)	0.0417 (5)	
H16A	0.6065	0.3437	0.1961	0.050*	
H16B	0.8265	0.4133	0.2251	0.050*	
C17	0.8749 (7)	0.4267 (3)	0.15168 (9)	0.0539 (7)	
H17A	0.9934	0.3593	0.1496	0.081*	
H17B	0.9749	0.5003	0.1525	0.081*	
H17C	0.7603	0.4270	0.1240	0.081*	

### Atomic displacement parameters (Å^2^)

**Table:**

	*U*^11^	*U*^22^	*U*^33^	*U*^12^	*U*^13^	*U*^23^
O1	0.0414 (11)	0.0677 (13)	0.0635 (12)	0.0013 (10)	0.0107 (10)	0.0136 (11)
O2	0.0805 (16)	0.0535 (11)	0.0528 (10)	−0.0074 (11)	0.0050 (12)	0.0097 (10)
O3	0.0437 (10)	0.0448 (9)	0.0465 (9)	0.0102 (8)	0.0082 (9)	0.0071 (8)
O4	0.0578 (12)	0.0402 (8)	0.0519 (10)	0.0089 (8)	0.0092 (10)	0.0100 (8)
N1	0.0411 (12)	0.0485 (11)	0.0439 (11)	−0.0042 (9)	0.0058 (10)	0.0092 (9)
N2	0.0441 (13)	0.0508 (11)	0.0445 (11)	−0.0076 (10)	0.0024 (11)	0.0059 (10)
C1	0.0379 (13)	0.0411 (11)	0.0380 (12)	−0.0011 (10)	−0.0006 (10)	−0.0033 (9)
C2	0.0437 (15)	0.0510 (13)	0.0461 (13)	0.0085 (11)	0.0032 (12)	−0.0063 (12)
C3	0.0602 (18)	0.0456 (13)	0.0496 (15)	0.0083 (13)	−0.0036 (14)	0.0001 (12)
C4	0.0525 (16)	0.0438 (12)	0.0416 (12)	−0.0070 (12)	−0.0032 (12)	−0.0025 (11)
C5	0.0522 (17)	0.0563 (14)	0.0462 (14)	0.0015 (13)	0.0104 (13)	0.0041 (12)
C6	0.0467 (14)	0.0447 (12)	0.0500 (14)	0.0076 (11)	0.0051 (13)	−0.0009 (11)
C7	0.0383 (13)	0.0431 (11)	0.0431 (12)	−0.0021 (10)	0.0031 (12)	−0.0028 (10)
C8	0.0413 (14)	0.0457 (12)	0.0448 (13)	−0.0052 (11)	0.0028 (11)	0.0008 (10)
C9	0.0387 (13)	0.0383 (10)	0.0411 (12)	−0.0036 (9)	−0.0027 (11)	0.0007 (10)
C10	0.0353 (13)	0.0359 (10)	0.0448 (12)	0.0015 (9)	−0.0023 (10)	0.0017 (9)
C11	0.0321 (11)	0.0354 (10)	0.0415 (11)	−0.0018 (9)	0.0004 (10)	−0.0005 (9)
C12	0.0350 (12)	0.0314 (9)	0.0457 (12)	0.0003 (9)	−0.0014 (11)	0.0010 (9)
C13	0.0419 (14)	0.0344 (11)	0.0549 (14)	0.0030 (10)	0.0051 (13)	−0.0016 (10)
C14	0.0430 (14)	0.0454 (12)	0.0480 (13)	−0.0020 (11)	0.0075 (12)	−0.0017 (11)
C15	0.069 (2)	0.099 (3)	0.0512 (16)	−0.021 (2)	0.0028 (18)	0.0182 (18)
C16	0.0397 (13)	0.0379 (11)	0.0474 (13)	0.0053 (10)	0.0016 (12)	0.0000 (10)
C17	0.0583 (18)	0.0576 (15)	0.0460 (14)	0.0112 (14)	0.0044 (14)	−0.0024 (12)

### Geometric parameters (Å, º)

**Table:**

O1—C7	1.229 (3)	C6—H6A	0.9300
O2—C4	1.373 (3)	C8—C9	1.455 (3)
O2—C15	1.431 (5)	C8—H8A	0.9300
O3—C11	1.366 (3)	C9—C14	1.390 (4)
O3—C16	1.443 (3)	C9—C10	1.400 (4)
O4—C12	1.358 (3)	C10—C11	1.385 (3)
O4—H1O4	0.8198	C10—H10A	0.9300
N1—C7	1.356 (4)	C11—C12	1.403 (3)
N1—N2	1.386 (3)	C12—C13	1.386 (4)
N1—H1N1	0.8977	C13—C14	1.389 (3)
N2—C8	1.286 (4)	C13—H13A	0.9300
C1—C6	1.396 (4)	C14—H14A	0.9300
C1—C2	1.398 (4)	C15—H15A	0.9600
C1—C7	1.481 (3)	C15—H15B	0.9600
C2—C3	1.368 (4)	C15—H15C	0.9600
C2—H2A	0.9300	C16—C17	1.501 (4)
C3—C4	1.388 (4)	C16—H16A	0.9700
C3—H3A	0.9300	C16—H16B	0.9700
C4—C5	1.384 (4)	C17—H17A	0.9600
C5—C6	1.385 (4)	C17—H17B	0.9600
C5—H5A	0.9300	C17—H17C	0.9600
			
C4—O2—C15	117.6 (3)	C11—C10—C9	120.1 (2)
C11—O3—C16	116.72 (18)	C11—C10—H10A	119.9
C12—O4—H1O4	109.3	C9—C10—H10A	119.9
C7—N1—N2	117.9 (2)	O3—C11—C10	125.8 (2)
C7—N1—H1N1	120.2	O3—C11—C12	114.3 (2)
N2—N1—H1N1	115.3	C10—C11—C12	119.9 (2)
C8—N2—N1	114.5 (2)	O4—C12—C13	118.3 (2)
C6—C1—C2	117.8 (2)	O4—C12—C11	121.8 (2)
C6—C1—C7	123.4 (2)	C13—C12—C11	119.9 (2)
C2—C1—C7	118.8 (2)	C12—C13—C14	119.9 (2)
C3—C2—C1	120.9 (3)	C12—C13—H13A	120.0
C3—C2—H2A	119.5	C14—C13—H13A	120.0
C1—C2—H2A	119.5	C13—C14—C9	120.6 (2)
C2—C3—C4	120.5 (3)	C13—C14—H14A	119.7
C2—C3—H3A	119.8	C9—C14—H14A	119.7
C4—C3—H3A	119.8	O2—C15—H15A	109.5
O2—C4—C5	124.2 (3)	O2—C15—H15B	109.5
O2—C4—C3	115.8 (3)	H15A—C15—H15B	109.5
C5—C4—C3	120.1 (3)	O2—C15—H15C	109.5
C4—C5—C6	119.1 (3)	H15A—C15—H15C	109.5
C4—C5—H5A	120.5	H15B—C15—H15C	109.5
C6—C5—H5A	120.5	O3—C16—C17	107.8 (2)
C5—C6—C1	121.7 (3)	O3—C16—H16A	110.2
C5—C6—H6A	119.2	C17—C16—H16A	110.2
C1—C6—H6A	119.2	O3—C16—H16B	110.2
O1—C7—N1	122.6 (2)	C17—C16—H16B	110.2
O1—C7—C1	122.4 (3)	H16A—C16—H16B	108.5
N1—C7—C1	115.0 (2)	C16—C17—H17A	109.5
N2—C8—C9	122.2 (2)	C16—C17—H17B	109.5
N2—C8—H8A	118.9	H17A—C17—H17B	109.5
C9—C8—H8A	118.9	C16—C17—H17C	109.5
C14—C9—C10	119.5 (2)	H17A—C17—H17C	109.5
C14—C9—C8	118.6 (2)	H17B—C17—H17C	109.5
C10—C9—C8	122.0 (2)		
			
C7—N1—N2—C8	−172.3 (2)	N1—N2—C8—C9	−178.2 (2)
C6—C1—C2—C3	1.4 (4)	N2—C8—C9—C14	−162.0 (3)
C7—C1—C2—C3	−179.0 (3)	N2—C8—C9—C10	18.0 (4)
C1—C2—C3—C4	−1.6 (4)	C14—C9—C10—C11	0.1 (4)
C15—O2—C4—C5	10.0 (4)	C8—C9—C10—C11	−179.9 (2)
C15—O2—C4—C3	−170.4 (3)	C16—O3—C11—C10	3.6 (3)
C2—C3—C4—O2	−179.4 (3)	C16—O3—C11—C12	−175.6 (2)
C2—C3—C4—C5	0.3 (4)	C9—C10—C11—O3	−177.6 (2)
O2—C4—C5—C6	−179.2 (3)	C9—C10—C11—C12	1.6 (4)
C3—C4—C5—C6	1.2 (4)	O3—C11—C12—O4	−1.8 (3)
C4—C5—C6—C1	−1.3 (4)	C10—C11—C12—O4	178.9 (2)
C2—C1—C6—C5	0.1 (4)	O3—C11—C12—C13	177.1 (2)
C7—C1—C6—C5	−179.5 (3)	C10—C11—C12—C13	−2.2 (4)
N2—N1—C7—O1	0.8 (4)	O4—C12—C13—C14	180.0 (2)
N2—N1—C7—C1	−178.3 (2)	C11—C12—C13—C14	1.0 (4)
C6—C1—C7—O1	150.6 (3)	C12—C13—C14—C9	0.7 (4)
C2—C1—C7—O1	−29.0 (4)	C10—C9—C14—C13	−1.2 (4)
C6—C1—C7—N1	−30.2 (4)	C8—C9—C14—C13	178.8 (2)
C2—C1—C7—N1	150.2 (2)	C11—O3—C16—C17	178.5 (2)

### Hydrogen-bond geometry (Å, º)

*Cg*1 is the centroid of the C9–C14 ring.

**Table:**

*D*—H···*A*	*D*—H	H···*A*	*D*···*A*	*D*—H···*A*
O4—H1*O*4···O3	0.82	2.41	2.683 (2)	100
O4—H1*O*4···O1^i^	0.82	2.21	2.981 (2)	156
N1—H1*N*1···O1^ii^	0.90	2.10	2.994 (3)	172
C10—H10*A*···O4^iii^	0.93	2.55	3.462 (3)	168
C16—H16*B*···*Cg*1^i^	0.97	2.68	3.499 (2)	142

Symmetry codes: (i) −*x*+1, *y*+1/2, −*z*+1/2; (ii) *x*−1, *y*, *z*; (iii) −*x*+1, *y*−1/2, −*z*+1/2.
